# Bitter melon protects against ER stress in LS174T colonic epithelial cells

**DOI:** 10.1186/s12906-016-1522-1

**Published:** 2017-01-03

**Authors:** Dale A. Kunde, Wai Chin Chong, Prathiba V. Nerurkar, Kiran D.K. Ahuja, Jeremy Just, Jason A. Smith, Nuri Guven, Rajaraman D. Eri

**Affiliations:** 1 School of Health Sciences, University of Tasmania, Launceston, 7250 TAS Australia; 2 Department of Molecular Biosciences & Bioengineering, University of Hawaii, Honolulu, HI USA; 3 School of Physical Sciences, University of Tasmania, Hobart, 7000 TAS Australia; 4 School of Medicine, University of Tasmania, Hobart, 7000 TAS Australia; 5 School of Health Sciences, University of Tasmania, Locked Bag 1322, Launceston, TAS 7250 Australia

**Keywords:** Inflammatory bowel disease, Intestinal secretory cells, Endoplasmic reticulum stress, Oxidative stress, Unfolded protein responses, Bitter melon

## Abstract

**Background:**

Bitter Melon (BM) has been used as a functional food in traditional Chinese and Indian medicine for many generations and has gained a great deal of attention due to its apparent benefits in moderating some of the pathogenic processes in a variety of inflammatory conditions. BM extract (BME) has been shown to possess strong anti-oxidant properties. In addition, it can ameliorate oxidative stress and potentially ER stress. There is increasing evidence that oxidative and ER stress are major contributors for intestinal secretory cell dysfunction which leads to local inflammation and disease pathogenesis that are hallmarks of inflammatory bowel diseases (IBD). Hence, the search for potential therapeutics against ER stress and oxidative stress in intestinal epithelial secretory cells may provide valuable resources for the management of IBD. The aim of the present study was to investigate the effects of BME in ameliorating ER stress in colonic epithelial cells.

**Methods:**

Human colonic adenocarcinoma LS174T cells were used for the assessment of BME effects on colonic epithelial cells in vitro. Cell viability was assessed using trypan blue exclusion and the effect of BME in ameliorating tunicamycin (TM)-induced ER stress was determined by analysing the mRNA expression of the common ER stress markers; ATF6, XBP1, GRP78, CHOP and PERK by quantitative RT-PCR and GRP78 and CHOP by western blot.

**Results:**

In the absence of ER stress, BME exhibited no cell toxicity up to 2.0% w/v and no significant effect on the basal mRNA expression of ER stress markers in LS174T cells. In contrast, pre-treatment of LS174T cells with BME followed by induction of ER stress resulted in a significant decrease in mRNA expression of ATF6, XBP1, GRP78, CHOP and PERK and protein expression of GRP78 and CHOP. Co-treatment during induction of ER stress and post- treatment following induction of ER Stress in LS174T cells resulted in a lower but still significant reduction in mRNA expression levels of most ER stress markers.

**Conclusions:**

This is one of the first studies demonstrating the efficacy of BME in reducing expression of ER stress markers in colonic epithelial cells suggesting the potential of BME as a dietary intervention in ameliorating ER stress and oxidation in IBD. Interestingly, while the most significant effect was seen with pre-treatment of cells with BME there was a reduced but still significant effect when co-treated or even post-treated. This suggests that BME may even be effective in modulating ER stress in the face of an existing cell stress environment.

**Electronic supplementary material:**

The online version of this article (doi:10.1186/s12906-016-1522-1) contains supplementary material, which is available to authorized users.

## Background

Bitter melon (BM; *Momordica charantia*), also known as bitter gourd, karela or balsam pear, belongs to the *Cucurbitaceae* family and is widely cultivated in tropical regions including Asia, Africa and South America. It has been used in traditional Chinese and Indian medicines for gastrointestinal disorders as well as diabetes and its complications [1–4]. Extensive characterization of BM (for review see [5]) has identified various bioactive components of BM such as kuguacin J, karaviloside XI, kuguaglycoside C, momordicoside Q-U, charantin, vicine, polypeptides and a variety of polyphenols and proteins that are thought to contribute to its beneficial effects [3, 5–7]. BM has gained worldwide attention due to its apparent diverse physiological benefits such as hypolipidaemia, hypoglycaemia, anti-viral, anti-inflammatory, anti-cancer properties as well as strong antioxidant properties [7–12]. However, little is known about the effects of BM on ER-stress related inflammatory conditions and its potential benefits in these conditions. Inflammatory bowel disease (IBD) is characterised by a chronic and exaggerated inflammatory immune response to the intestinal microbial flora influenced by a complex interaction of genetic predisposition, environmental triggers and dysregulated immune system, which comprise primarily Crohn’s disease (CD) and ulcerative colitis (UC) [13, 14]. Intestinal barrier dysfunction due to defects in the intestinal secretory cells as a result of increased oxidative and ER stress has been recognised as a major contributor to the pathogenesis of IBD [13, 15].

Several mouse models such as *Winnie*, *Eeyore* and *Kenny* (missense mutations in MUC2) and Agr2 deficiency (loss of MUC2 production) illustrate the link between intestinal epithelial cell ER stress with intestinal inflammation [13, 16]. Elevated levels of oxidative stress in intestinal secretory cells as a consequence of ER stress leads to the depletion of intestinal mucins, enhanced mucosal permeability and extensive disruption of the intestinal epithelial cells and results in spontaneous intestinal inflammation [17, 18]. We used LS174T colonic epithelial cells as they have the highest expression of MUC2 mucin, the major mucin that fills human colonic lumen and hence a better representation of colonic goblet cells that have the propensity for ER stress.

Several mechanisms have been proposed to explain the connection between ER stress and oxidative stress [19]. The formation of intra and inter-molecular disulphide bonds through oxidation involving two major enzymes, protein disulphide isomerase (PDI) and ER oxidoreductin 1 (ERO1) are fundamental to the protein folding process. This process requires significant energy through oxidation of cysteine residues and electron transfer. An excess protein-folding load can result in the accumulation of ROS triggering a cellular inflammatory response. Increased consumption of glutathione (GSH) to repair the unfolded or mis-folded proteins reduces available cellular GSH in the cells, leading to the initiation of the unfolded protein response (UPR), which is a signature of ER stress [20].

With a developing understanding of the pathogenesis of IBD, various therapeutic strategies that ameliorate ER stress and oxidative stress in IBD have been introduced. Examples include, Infliximab; a mAb targeting tumour necrosis factor (TNF) to suppress TNF-mediated inflammatory cytokine release, Salubrinal, an eIF2-α dephosphorylation inhibitor protecting cells from ER stress induced apoptosis, and more recently, Dexamethasone (DEX); suppressing ER stress and UPR activation in both in vitro and in vivo studies [13, 21–23].

Furthermore, the potential of antioxidant therapeutic drugs such as sulfasalazine, immunosuppressive agents, steroids, iron chelators and N-acetylcysteine (NAC) have been shown to be beneficial in IBD patients [17, 24]. However, as a result of the high health-care costs and the presence of drug side effects, there is increased attention towards the use of functional foods as a possible therapeutic approach for treating, or even potentially minimising the risk of developing IBD [3, 25, 26]. Although numerous studies have shown the extensive beneficial effects of BM in a variety of conditions, little is known regarding the effect of BM towards ameliorating ER stress and oxidative stress in colonic epithelial cells.

The aim of this study was to investigate the potential of BM extract (BME) to ameliorate TM-induced ER stress in colonic epithelial cells.

## Methods

### Preparation of BME by pressurised hot water extraction

Active ingredients were extracted from BM (Chinese variety; sourced from local vegetable super market in Hobart, Tasmania, Australia labelled as bitter melon) by pressurised hot water using a modification of a method previously described for natural product isolation [27, 28].

Fresh whole BM was sliced and dried in an oven at 35 °C until brittle. The slices were ground to a coarse powder and 4 g of this preparation was mixed with 12 g of acid washed sand followed by packing into the filter basket of a household espresso coffee machine.

Approximately 200 mL of deionised water was passed through the sample and the extract was evaporated to dryness by rotary evaporation under vacuum at 50 °C. Typical yields were 1 g BME per 4 g dehydrated BM. Working BME used for the in vitro studies was prepared by dissolving 1.0 g of dried BME in 50 mL of MilliQ grade water with periodic vortexing over 15 min. The extract was centrifuged at 560 × g at 4 °C for 30 min to remove solids providing a BME of 20% (w/v) which was stored at −20 °C for later use. Stock BME was diluted to the desired concentration in PBS for subsequent experiments.

### LC-MS Analysis of BME

Aqueous samples of BME prepared above were analysed using a Waters Acquity H-Class UPLC instrument coupled to a Waters Xevo triple quadrupole mass spectrometer. A Waters Acquity UPLC BEH C18 column (2.1 mm × 100 mm × 1.7 μm) was used. The mobile phase consisted of two solvents: 10 mM Ammonium Formate in water (solvent A) and Acetonitrile (solvent B). The UPLC program was initially 100% A, followed by a linear gradient to 30% A at 4.0 min and 10% A at 6 min, which was held for 2 min before returning to initial conditions and re-equilibration for 3 min. The flow rate was 0.35 mL/min, the column was held at 35 °C, and the sample compartment was at 6 °C. The mass spectrometer was operated in positive ion electrospray mode with a needle voltage of 2.6 kV, a desolvation gas (Nitrogen) flow of 950 L/hr at 450 °C and cone gas flow of 50 L/hr. Full scan mass spectrometry experiment was conducted over the range (m/z) 150 to 1150 with a cone voltage of 32 V and scan time of 0.5 sec.

### Antioxidant activity of BME

The anti-oxidant activity of BME was assessed by its effect on the copper-induced oxidation of human serum. Fasting plasma was collected from 6 healthy individuals (three men and three women) and isolated serum was frozen at −80 °C prior to analysis of BME serum oxidation. Copper-induced oxidation of human serum was performed in duplicate using the method described previously [29, 30]. Serum was diluted 50-fold in phosphate buffered saline (pH 7.4), incubated with increasing concentrations of BME (0, 0.0625, 0.125, 0.25, 0.50 and 0.75%), and subjected to copper (50 μM**)-**induced oxidation. Oxidation kinetics were determined for each serum sample and concentrations of BME by using a multiposition spectrophotometer (Cintra 10E UV–vis, GBC Scientific Equipment, AUS) to measure the absorbance at 245 nm at 37 °C for every 10 min for 360 min.

### LS174T cell culture

Human colon carcinoma cells LS174T (ATCC) were cultured as an adherent monolayer in RPMI-1640 + Glutamax (Life Technologies, AUS), supplemented with 10% heat-inactivated FBS (Gibco BRL, AUS) and penicillin (1000 U/mL) and streptomycin (1000 ug/L) (Gibco BRL, AUS). Cultures were maintained at 37oC in a humidified 5% CO2 and media replaced and cells passaged every 2 to 4 days. For experimental procedures, cultures were grown to 80-95% confluence and harvested using 0.25% TrypLe Express (Life Technologies, AUS). Cells were washed twice in culture media and number and viability were assessed using the Countess® cell counter (Life Technologies, AUS) according to manufacturer’s instructions.

### Effect of BME on cell viability

To assess the effect of BME on cell viability of LS174T cells, 12-well cell culture plates (Greiner, AUS) were seeded at a density of 3.0 × 105 cells per well in 2.0 mL of medium and incubated overnight at 37oC/5% CO2 to allow the cells to adhere. The medium was replaced the next day with medium containing BME (0.5, 1.0, 1.5 and 2.0% w/v). After 6 h incubation, the cells were washed with HBSS and harvested by detaching the cells from the growth surface with TrypLe Express (Life Technologies, AUS), washed twice with HBSS and cell viability assessed using the Countess® cell counter (Life Technologies, AUS) according to manufacturer’s instructions.

### Baseline expression of ER markers in LS174T cells treated with BME

To assess the baseline expression of ER stress markers in LS174T cells treated with BME, 12-well cell culture plates (Greiner, AUS) were seeded at a density of 3.0 × 105 cells per well in 2.0 mL of medium and incubated overnight at 37oC/5% CO2 to allow the cells to adhere. The medium was replaced the next day with treatment medium containing BME (0.001, 0.01, 0.1, 0.5 and 1.0%) followed by incubation for 6 h at 37oC/5% CO2. Control cells were treated with PBS as vehicle controls. Total RNA was extracted and mRNA expression of ER stress markers assessed as described below.

### Effect of BME on the expression of ER stress markers in LS174T in response to induction of ER stress with TM

The effect of BME on TM-induced ER stress in LS174T cells was assessed in a pre, during and post treatment regimen. For mRNA expression analysis, 12-well cell culture plates were seeded at a density of 3.0 × 105 cells per well in 2.0 mL of medium and incubated overnight at 37oC/5% CO2 to allow the cells to adhere. For protein expression analysis, 30 mm cell-culture dishes (Greiner, AUS) were seeded with 1 × 106 of in 5.0 mL of medium and incubated overnight at 37oC/5% CO2 to allow the cells to adhere. For the pre-treatment regimen, the media was replaced the next day with medium containing BME (0, 0.125% and 0.25%) for 6 h at 37oC/5% CO2 after which media was replaced with fresh medium containing TM (10 μg/mL in DMSO) for a further 6 h at 37oC/5% CO2. For the co-treatment regimen, the media was replaced with medium containing BME (0, 0.125% and 0.25%) and TM (10 μg/mL in DMSO) for 6 h at 37oC/5% CO2. For the post-treatment regimen, the media was replaced with medium containing TM (10 μg/mL in DMSO) for 6 h at 37oC/5% CO2 after which media was replaced with fresh medium containing BME (0, 0.125% and 0.25%) for a further 6 h at 37oC/5% CO2. Control cells were treated with DMSO as a vehicle control at time points described. Total RNA and total protein was extracted and mRNA or protein expressions of ER stress markers were assessed as described below. Each treatment was performed in duplicate and the data presented are the mean ± SEM of 5 independent experiments.

### RNA extraction and cDNA synthesis

Total RNA was extracted from 3 × 10^5^ treated LS174T cells using RNeasy Mini Kit (Qiagen, AUS) following the manufacturer’s protocol including an on-column DNase digestion using the RNase-Free DNase set (Qiagen, AUS) to remove gDNA. RNA quality and quantity was assessed using the Experion automated electrophoresis system (Bio-Rad Laboratories, AUS) and RNA samples with an RQI > 7.0 were considered suitable for expression analysis. One microgram of total RNA was reverse transcribed to cDNA using the iScript cDNA synthesis kit (Bio-Rad Laboratories, AUS) following the manufacturer’s protocol.

### Quantitative real time polymerase chain reaction (qRT-PCR)

Quantitative RT-PCR was performed using predesigned Taqman® probe/primer assays (Life Technologies, AUS) for GAPDH (Hs03929097_gl), ATF6 (Hs00232586_m1), XBP1 (Hs00231936_m1), GRP78 (Hs0060719_gH), CHOP (Hs00358796_g1) and PERK (Hs00984006_m1). The qRT-PCR reactions contained 40 ng cDNA, TaqMan Fast Advanced Master Mix (Life Technologies, AUS), 1 μL of gene specific Taqman probe/primer mix in a total volume of 20 μL. Reactions were performed in duplicate on the StepOne Plus RT-PCR system (Life Technologies, AUS) with thermal cycling conditions of: an initial denaturation step at 95 °C for 20 s, followed by 40 cycles at 95 °C for 1 s and 60 °C for 20 s. The mean threshold cycle was determined for each sample and used for relative expression analysis.

### Western blot analysis of GRP78 and CHOP

Total cell protein was extracted from 1 × 10^6^ treated LS174T cells by firstly washing the cells with HBSS followed by homogenization in 2 mL of RIPA buffer/10% of Protease Inhibitor (Sigma-Aldrich, AUS) and then cleared by centrifugation at 12000 rpm for 20 min at 4 °C. Thirty micrograms of protein from each sample was denatured in Laemmli loading buffer (Bio-Rad Laboratories, AUS) and separated on precast 12% SDS-PAGE gels (Bio-Rad Laboratories, AUS) followed by overnight transfer onto PVDF membranes (Millipore, AUS) at 30 mV at 4 °C. The blot was blocked with 5% non-fat milk, before being incubated with anti-GADPH (#14C10, 1:3000, Novus Biologicals, AUS), anti-GRP78 (NBP-06274, 1:1000, Novus Biologicals, AUS) and anti-CHOP (NBP2-13172, 1:1000, Novus Biologicals, AUS) overnight at 4 °C in blocking buffer. The blot was washed in PBST and incubated with appropriate species monoclonal horseradish peroxidase-conjugated anti-IgG secondary antibodies (1:5000) for 1 h at 20 °C. Bands were visualized using the Supersignal West Pico chemiluminescent kit (Thermo Scientific, AUS), digitised and band intensities determined using a Fuji LAS-3000 Imager (Fuji Life Sciences, JPN). Samples from all groups were included in each individual blot to ensure accurate quantification across multiple blots.

### Data analysis

Copper-induced oxidation curves were plotted using PRISM v5 (GraphPad Software, San Diego, CA, USA) and antioxidant capacity of BME was assessed by calculating lag time, rate of oxidation and maximum change in absorbance for each curve. Lag time, an indicator of the protection of the serum against oxidation, was determined as the intercept between baseline (time zero) and the tangent of the absorbance curve during the propagation phase. The rate of oxidation was calculated as the slope of the propagation phase. The maximum change in absorbance was calculated by the difference between 0 min to 360 min for each BME concentration tested in each of the serum samples. The lag time and the rate of oxidation were calculated manually by blinding each concentration for each serum sample to avoid any bias.

Data are presented as mean ± SEM and significance testing achieved using two-way ANOVA followed by Games-Howell pairwise comparisons using SPSS v20.0 software (IBM, Armonk, NY, USA).

Relative mRNA expression for the ER stress markers and significance testing to controls was conducted using Relative Gene Expression (REST) 2009 software (Qiagen, AUS) based on the ΔΔCt method [31] where expression of each gene in each treatment group was expressed relative to the mean of a relevant vehicle control or treatment group as described and normalised to the housekeeping gene, GAPDH and significance testing achieved through a pair-wise fixed reallocation randomisation test. Data are presented as the mean mRNA fold change ± SEM and significance testing achieved using two-way ANOVA followed by Games-Howell pairwise comparisons using SPSS v20.0 software (IBM, Armonk, NY, USA).

Relative protein expression for ER stress markers was determined from band intensities and normalized using GADPH expression and calculated as relative expression over vehicle.

Data are presented as mean ± SEM and significance testing achieved using two-way ANOVA followed by Games-Howell pairwise comparisons using SPSS v20.0 software (IBM, Armonk, NY, USA).

## Results

### LC-MS analysis of BME yields a complex mix of constituents

**Fig. 1:**
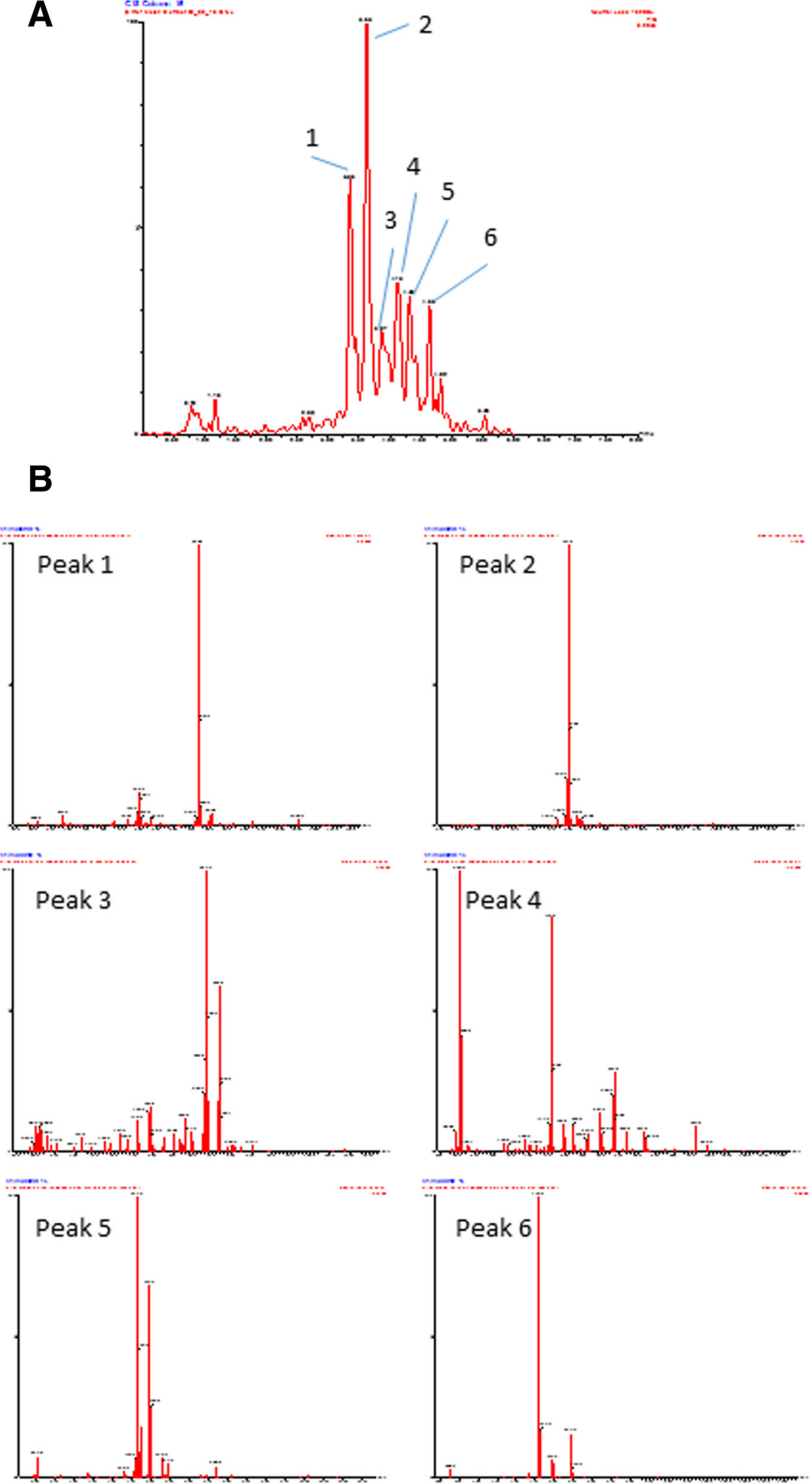
LC-MS analysis of steam extracted BME. **a** Constant Neutral Loss LC chromatogram using a Waters Acquity UPLC BEH C18 column and gradient Formate: Acetonitrile elution profile. **b** MS analysis of significant LC peaks (peaks 1–6) by full scan mass spectrometry conducted over the range (m/z) 150 to 1150 with a cone voltage of 32 V and scan time of 0.5 sec

LC-MS analysis of the bitter melon extract indicates a complex mixture and the potential for four major components to be present (Fig. 1a). Analysis of the mass spectra of these peaks was unable to be assigned to metabolites previously isolated, particularly the anti-diabetic triterpenoid glycosides reported previously (Fig. 1b) [32].

### BME possesses potent antioxidant capacity

We first analysed the anti-oxidant capacity of BME by determining its ability to ameliorate copper-induced oxidation of serum from healthy volunteers (Additional file 1 Figure S1). An increase in lag time of oxidation was observed with increasing concentrations of BME (Additional file 2 Table S1) where the lag time ranged from 144 ± 15 min for vehicle to >360 min for BME 0.75%. The lag times for all concentrations of BME at 0.125% BME or greater were significantly longer (*p* < 0.05) than the control. There was no significant change in the rate of oxidation with increasing concentrations of BME. The maximum change in absorbance ranged from 0.570 ± 0.035 for the vehicle to 0.490 ± 0.0.038 abs at BME 0.25% (Additional file 2 Table S1) but was not significantly different, while the maximum change in absorbance for the BME treatments of 0.5% and 0.75% were significantly lower (0.295 ± 0.045 and 0.079 ± 0.009 respectively; *p* < 0.05) due to incomplete oxidation reaction resulting from an increased lag time.

### Effect of BME treatment on cell viability and activation of UPR in LS174T cells

Increasing concentrations of BME up to 1.0% (w/v) for 6 h did not affect the viability of LS174T cells compared to vehicle, as determined by trypan blue exclusion (Data not shown).

The activation of UPR in LS174T cells treated with increasing concentrations of BME for 6 h was assessed by quantitative mRNA expression for ATF6, XBP1, GRP78 and CHOP.

**Fig. 2:**
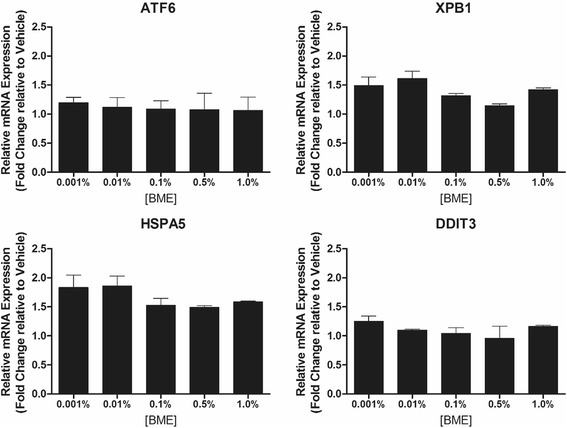
mRNA expression of UPR genes ATF6, XBP1 GRP78 and CHOP in LS174T cells treated with BME 0.001 –1.00% for 6 h were measured by qRT-PCR and relative mRNA expression levels are vs control and normalised to *GAPDH* (*n* = 3). Data are shown as the mean fold change ± SEM (vs vehicle: ^*^, ^**^, ^***^; *p* < 0.05, *p* < 0.01 and *p* < 0.001 respectively)

Compared to vehicle-treated cells no significant dose-dependent effects of BME on the mRNA expression levels of any of the markers were detected in LS174T cells (Fig. 2).

### Effect of pre, co and post treatment using BME on TM-induced ER stress in LS174T cells

**Fig. 3:**
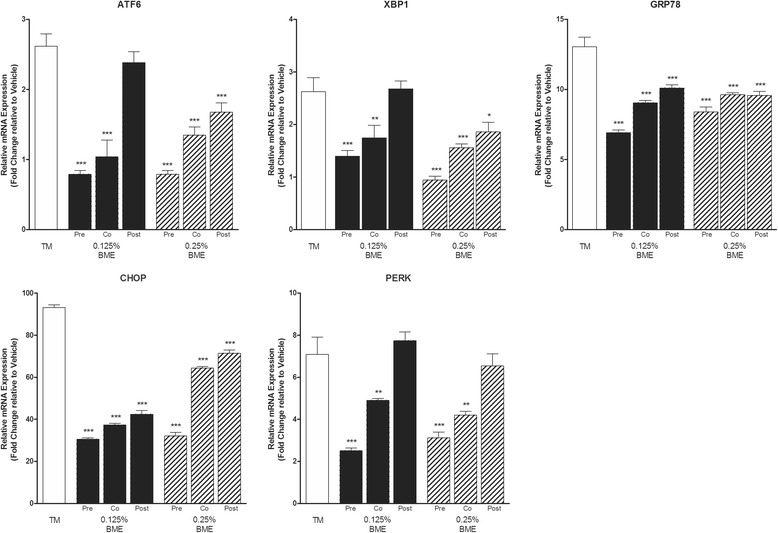
BME suppresses TM-induced UPR activation in LS174T colonic epithelial cells. LS174T cells pre-treated, co-treated or post-treated with 0.125% and 0.25% BME for 6 h prior to, during or after ER stress induction using tunicamycin (10 μg/mL). mRNA expression of UPR genes ATF6, XBP1, GRP78, CHOP and PERK in LS174T cells were measured by qRT-PCR. The mRNA gene expression is relative to TM (10ug/mL in DMSO) expression and normalized to GAPDH (*n* = 5) Data are shown as the mean fold change ± SEM (significance testing vs TM: ^*^, ^**^, ^***^; *p* < 0.05, *p* < 0.01 and *p* < 0.001 respectively)

**Fig. 4:**
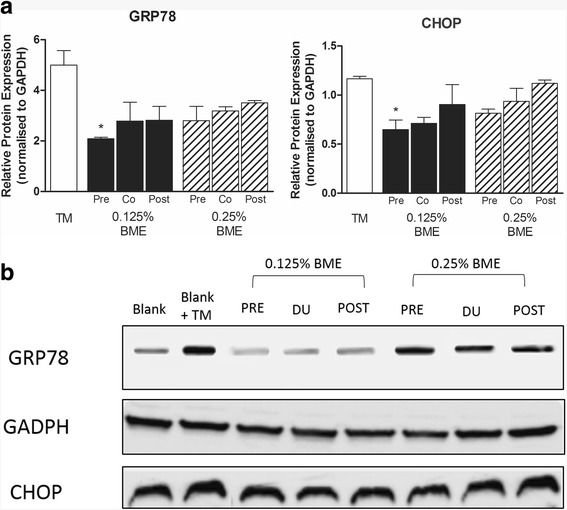
Western blot analysis of ER Stress markers in LS174T cells. **a**: Representative western blot. **b**: Cellular protein expression for GRP78 and CHOP in LS174T cells pre-treated, co-treated or post-treated with 0.125% and 0.25% BME for 6 h prior to, during or after ER stress induction using tunicamycin (10 ug/mL). The band densities were normalized to GAPDH band density. Data shown as means ± SEM (*n* = 2) (significance testing vs TM: *, **, ***; *p* < 0.05, *p* < 0.01 and *p* < 0.001 respectively)

We next investigated the efficacy of BME in ameliorating ER stress in LS174T cells. Exposure to tunicamycin (10 μg/mL) for 6 h resulted in a significantly increased mRNA expression of all ER stress markers relative to vehicle treated cells; ATF6 (2.62 ± 0.18 fold; 284 *p* < 0.05), XBP1 (2.6 ± 0.26 fold; *p* < 0.05), GRP78 (13.0 ± 0.7 fold; *p* < 0.01), CHOP (93.2 ± 1.28 fold; *p* < 0.001) and PERK (7.12 ± 0.83; *p* < 0.01) (mean ± SEM; *n* = 5) corresponding to ER stress induction. Pre-treatment of LS174T cells with 0.125% or 0.25% BME for 6 h followed by induction of ER stress by exposure to tunicamycin (10 ug/mL) for a further 6 h significantly reduced the mRNA expression of all markers at both concentrations of BME tested (Fig. 3) indicating that BME suppresses TM-induced ER Stress in LS174T cells. ATF6 and CHOP were suppressed 3.3 and 3.1 fold respectively by pre-treatment with 0.125% BME while PERK, XBP1 and GRP78 which were suppressed 2.8, 1.9 and 1.9 fold respectively. Interestingly, both co-treatment and post-treatment of LS174T cells during and after TM-induced ER stress respectively also reduced the expression of most ER-stress markers but to a lesser extent with no significant affect seen for ATF6 and PERK for the post-treatment regimen (Fig. 3). Similar responses to BME were also seen in the cellular protein levels of GRP78 and CHOP as shown by western blot analysis (Fig. 4) confirming that modulation of genetic expression by BME is reflected in active protein suppression.

## Discussion

In this study, we have for the first time described the ability of BME in ameliorating ER stress in LS174T colonic epithelial cells. BME appears to moderate ER stress via a mechanism that reduces the expression of the ER stress marker CHOP expression more than other ER stress markers.

Detailed analysis of the constituents of BM typically yield a complex array of potentially bioactive compounds which have been reviewed comprehensively [5]. Our LC-MS analysis of the steam extractable components of BM used in this study also showed a complex mixture of components, though six major peaks were detected. However, mass spectra analysis of these peaks failed to allow specific designation to any isolated metabolites identified in previous studies. This highlights the complexity of identifying individual compounds that have potential beneficial effects and equally ignores any potential synergistic effects that may be seen.

Therefore, large scale extraction/isolation is warranted to determine metabolites responsible for the activity seen in this study. The beneficial effects of BM have been demonstrated in numerous pre-clinical studies. Mice fed with a high fat diet (HFD) supplemented with 0.5%, 0.75% or higher concentration of freeze-dried BME demonstrated an improvement in their glucose, lipid and lipoprotein metabolism [4, 33]. Preliminary studies have shown that BM reduces hepatic ER stress markers such as GRP78 and CHOP in HFD-fed mice [34]. BM also reduced the release of pro-inflammatory cytokines and inhibited neuroinflammation in the brains of HFD-fed mice [34].

Accumulation of mis-folded protein in TM-induced ER stress results in activation of the UPR via multiple intracellular signalling pathways that aim to restore ER homeostasis [23, 35, 36]. In this study, we demonstrated that BME significantly suppressed TM-induced ER stress in LS174T cells. This is supported by our results that demonstrated a decrease in UPR-related gene expression of ATF6, XBP1, GRP78, CHOP and PERK in LS174T cells when treated with BME 0.125% (w/v) and BME 0.25% (w/v). This suggests that dietary BME could be a potential therapeutic strategy in ameliorating ER stress in colonic epithelial cells in order to reduce the risk of IBD and also various ER stress-mediated human diseases that involve protein mis-folding. Interestingly, the modulation of ER stress markers appears to occur when LS174T cells are co-treated with BME during a current TM-induced ER stress period but also has an effect in suppressing markers after a TM-induced ER stress period, though to a lower degree.

This would suggest that BME would be effective in ameliorating cellular responses in an already stressed environment. However, surprisingly, 0.25% BME had less of an effect on cellular protein levels of GRP78 and CHOP compared to 0.125% BME. The reason for this effect could be due to the competing activities within the extract are likely the cause of the absence of a clear dose response at the level of CHOP expression, which warrants the separation of BME to identify the active components responsible for its multiple activities.

CHOP has been implicated as one of the most important components in the ER-stress induced apoptotic pathways that aims to maintain tissue homeostasis [37]. Increasing evidence suggests that dysregulation of apoptosis is a primary mechanism in various human diseases.

**Fig. 5:**
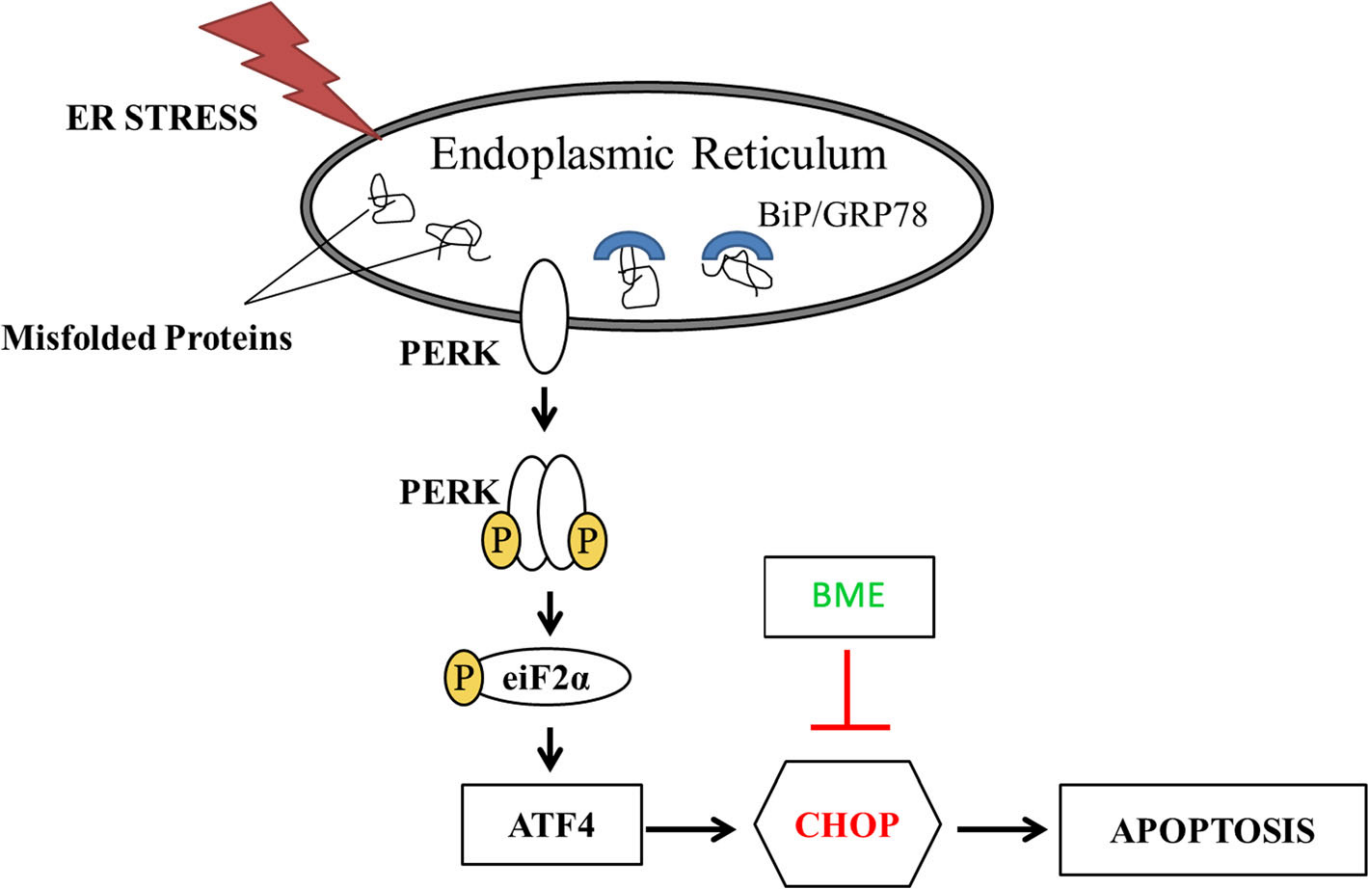
Proposed mechanism of BME ameliorating ER stress. BME ameliorates ER stress by supressing the expression of CHOP, a downstream target of PERK-eIF2α-ATF4 pathway from triggering apoptosis in the cells. ATF4, Active transcription factor 4

Therefore, targeting CHOP could be important in ameliorating ER stress [37]. Based on the findings of this study, we demonstrated that BME efficiently reduces ER stress processes involving CHOP, suggesting that the potential mechanisms in which BME ameliorates ER stress is by inhibiting the cells from apoptosis and providing cell viability that attempts to alleviate stress for survival (Fig. 5).

To our knowledge, this is the first study to demonstrate the effect of BME treatment on ER stress in colonic epithelial cells. Hence, this suggests that functional foods that contain bioactive compounds can potentially play a significant role in the protection of cells from oxidative damage due to increase intracellular ROS in the system as a result of ER stress [17, 38].

## Conclusions

In this study, we have demonstrated that BME effectively ameliorates ER stress in colonic epithelial cells. As ER stress and oxidative stress have been implicated in the pathogenesis of various diseases including IBD and based on these findings in this study, BME should be considered as a potential functional food in improving intestinal health by ameliorating ER stress and oxidative stress in IBD.

Further studies are warranted to investigate the active components of BME involved in ameliorating ER- and oxidative- stress in colonic epithelial cells, and to examine the effects of bioactive components in human colon cell lines. Determining the mechanisms of action of BME in the UPR pathways could be another interesting area to work on. Further in vivo studies could be done to determine the potential therapeutic effect of BM for the management of IBD.

## Additional files

Additional file 1: Figure S1. Copper-induced oxidation curves of human serum samples with increasing concentrations of BME w/v (▲; Control, ▼; 0.0625%; ●; 0.125%; **x**; 0.25%; ♦; 0.5% and ■; 0.75%). Data are shown as mean ± SEM of *n* = 6. (TIF 135 kb)
Additional file 2: Table S1. Effect of increasing concentrations of BME on copper-induced oxidation of human serum. (DOCX 12 kb)

## Abbreviations

ATF6: Activating transcription factor 6
BM: Bitter melon
BME: Bitter melon extract
CDL: Crohn’s disease
CHOP: CCAAT/-enhancer-binding protein homologous protein
ER: Endoplasmic reticulum
ERO1: ER oxidoreductin 1
GRP78: Glucose responsive protein 78
MUC2: Mucin 2
PERK: PRKR-like ER kinase
PDI: Protein disulphide isomerase
TM: Tunicamycin
UPR: Unfolded protein response
TNF: Tumor necrosis factor
XBP1: X-box binding factor 1

## References

[1] Anant S. Methanolic extracts of bitter melon inhibit colon cancer stem cells by affecting energy homeostasis and autophagy. Evid Based Complement Altern Med. 2013;2013. 10.1155/2013/702869 PMC3606719 23533514

[2] Kobori M, Nakayama H, Fukushima K, Ohnishi-Kameyama M, Ono H, Fukushima T, Akimoto Y, Masumoto S, Yukizaki C, Hoshi Y (2008). Bitter gourd suppresses lipopolysaccharide-induced inflammatory responses. J Agric Food Chem.

[3] Nerurkar P, Ray RB (2010). Bitter melon: antagonist to cancer. Pharm Res.

[4] Nerurkar PV, Pearson L, Efird JT, Adeli K, Theriault AG, Nerurkar VR (2005). Microsomal Triglyceride Transfer Protein Gene Expression and ApoB Secretion Are Inhibited by Bitter Melon in HepG2 Cells. J Nutr.

[5] Dandawate PR, Subramaniam D, Padhye SB, Anant S (2016). Bitter melon: a panacea for inflammation and cancer. Chin J Nat Med.

[6] Horax R, Hettiarachchy N, Islam S (2005). Total phenolic contents and phenolic acid constituents in 4 varieties of bitter melons (Momordica charantia) and antioxidant activities of their 401 extracts. J Food Sci.

[7] Lii C-K, Chen H-W, Yun W-T, Liu K-L (2009). Suppressive effects of wild bitter gourd (Momordica charantia Linn. var. abbreviata ser.) fruit extracts on inflammatory responses in RAW 264.7 macrophages. J Ethnopharmacol.

[8] Huang H-L, Hong Y-W, Wong Y-H, Chen Y-N, Chyuan J-H, Huang C-J, Chao P-M (2008). Bitter melon (Momordica charantia L.) inhibits adipocyte hypertrophy and down regulates lipogenic gene expression in adipose tissue of diet-induced obese rats. Brit J Nutr.

[9] Kubola J, Siriamornpun S (2008). Phenolic contents and antioxidant activities of bitter gourd (Momordica charantia L.) leaf, stem and fruit fraction extracts in vitro. Food Chem.

[10] Omara EA, Kam A, Alqahtania A, Li KM, Razmovski-Naumovski V, Nammi S, Chan K, Roufogalis BD, Li GQ (2010). Herbal medicines and nutraceuticals for diabetic vascular complications: mechanisms of action and bioactive phytochemicals. Curr Pharm Des.

[11] Wu S-J, Ng L-T (2008). Antioxidant and free radical scavenging activities of wild bitter melon (Momordica charantia Linn. var. abbreviata Ser.) in Taiwan. LWT-Food Sci Technol.

[12] Nerurkar PV, Johns LM, Buesa LM, Kipyakwai G, Volper E, Sato R, Shah P, Feher D, Williams PG, Nerurkar VR (2011). Momordica charantia (bitter melon) attenuates high-fat diet-associated oxidative stress and neuroinflammation. J Neuroinflammation.

[13] Das I, Png CW, Oancea I, Hasnain SZ, Lourie R, Proctor M, Eri RD, Sheng Y, Crane DI, Florin TH (2013). Glucocorticoids alleviate intestinal ER stress by enhancing protein folding and degradation of misfolded proteins. J Exp Med.

[14] Hanauer SB (2006). Inflammatory bowel disease: Epidemiology, pathogenesis, and therapeutic opportunities. Inflamm Bowel Dis.

[15] Kaser A, Blumberg R (2009). Endoplasmic reticulum stress and intestinal inflammation. Mucosal Immunol.

[16] Heazlewood CK, Cook MC, Eri R, Price GR, Tauro SB, Taupin D, Thornton DJ, Png CW, Crockford TL, Cornall RJ (2008). Aberrant mucin assembly in mice causes endoplasmic reticulum stress and spontaneous inflammation resembling ulcerative colitis. PLoS Med.

[17] Damiani CR, Benetton CA, Stoffel C, Bardini KC, Cardoso VH, Di Giunta G, Pinho RA, Dal‐Pizzol F, Streck EL (2007). Oxidative stress and metabolism in animal model of colitis induced by dextran sulfate sodium. J Gastroenterol Hepatol.

[18] Rezaie A, Parker RD, Abdollahi M (2007). Oxidative stress and pathogenesis of inflammatory bowel disease: an epiphenomenon or the cause?. Digest Dis Sci.

[19] Alfadda AA, Sallam RM. Reactive oxygen species in health and disease. J Biomed Biotechnol. 2012;2012:936486. 10.1155/2012/936486 PMC3424049 22927725

[20] Malhotra JD, Kaufman RJ (2007). Endoplasmic reticulum stress and oxidative stress: a vicious cycle or a double-edged sword?. Antioxid Redox Signal.

[21] Deuring JJ, Peppelenbosch M, Kuipers E, vander CJ W, de Haar C (2011). Impeded protein folding and function in active inflammatory bowel disease. Biochem Soc T.

[22] Kucharzik T, Maaser C, Lügering A, Kagnoff M, Mayer L, Targan S, Domschke W (2006). Recent understanding of IBD pathogenesis: Implications for future therapies. Inflamm Bowel Dis.

[23] McGuckin MA, Eri RD, Das I, Lourie R, Florin TH (2010). ER stress and the unfolded protein response in intestinal inflammation. American journal of physiology Gastrointestinal and liver physiology.

[24] Lih-Brody L, Collier KP, Reddy GM, Cerchia R, Kahn E, Weissman GS, Katz S, McKinley MJ, Fisher SE, Mullin GE (1996). Increased oxidative stress and decreased antioxidant defenses in mucosa of inflammatory bowel disease. Digest Dis Sci.

[25] Aggarwal BB, Shishodia S (2006). Molecular targets of dietary agents for prevention and therapy of cancer. Biochem Pharmacol.

[26] Hao X, Yao A, Gong J, Zhu W, Li N, Li J (2012). Berberine ameliorates pro-inflammatory cytokine-induced endoplasmic reticulum stress in human intestinal epithelial cells in vitro. Inflammation.

[27] Just J, Deans BJ, Olivier WJ, Paull B, Bissember AC, Smith JA (2015). New method for the rapid extraction of natural products: efficient isolation of shikimic acid from star anise. Org Lett.

[28] Just J, Jordan TB, Paull B, Bissember AC, Smith JA (2015). Practical isolation of polygodial from Tasmannia lanceolata: a viable scaffold for synthesis. Org Biomol Chem.

[29] Schnitzer E, Pinchuk I, Bor A, Fainaru M, Samuni AM, Lichtenberg D (1998). Lipid oxidation in unfractionated serum and plasma. Chem Phys Lipids.

[30] Ahuja KD, Kunde DA, Ball MJ, Geraghty DP (2006). Effects of capsaicin, dihydrocapsaicin, and curcumin on copper-induced oxidation of human serum lipids. J Agric Food Chem.

[31] Pfaffl MW, Horgan GW, Dempfle L (2002). Relative expression software tool (REST©) for group-wise comparison and statistical analysis of relative expression results in real-time PCR. Nucleic Acids Res.

[32] Tan MJ, Ye JM, Turner N, Hohnen-Behrens C, Ke CQ, Tang CP, Chen T, Weiss HC, Gesing ER, Rowland A (2008). Antidiabetic activities of triterpenoids isolated from bitter melon associated with activation of the AMPK pathway. Chem Biol.

[33] Chen Q, Chan LL, Li ET (2003). Bitter melon (Momordica charantia) reduces adiposity, lowers serum insulin and normalizes glucose tolerance in rats fed a high fat diet. J Nutr.

[34] Nerurkar PV. 2nd World Congress on. Diabetes. 2011;6(8).

[35] Bogaert S, De Vos M, Olievier K, Peeters H, Elewaut D, Lambrecht B, Pouliot P, Laukens D (2011). Involvement of endoplasmic reticulum stress in inflammatory bowel disease: a different implication for colonic and ileal disease?. PLoS One.

[36] Rasheva V, Domingos P (2009). Cellular responses to endoplasmic reticulum stress and apoptosis. Apoptosis.

[37] Oyadomari S, Mori M (2003). Roles of CHOP/GADD153 in endoplasmic reticulum stress. Cell Death Differ.

[38] Bhardwaj P (2010). Oxidative stress and antioxidants in gastrointestinal diseases. Trop Gastroenterol.

