# Supplementary figures and images for: Bitter melon protects against ER stress in LS174T colonic epithelial cells
Source: BMC Complement Altern Med. 2017 Jan 3;17:2. doi: 10.1186/s12906-016-1522-1 (PMC5210302; doi:10.1186/s12906-016-1522-1)

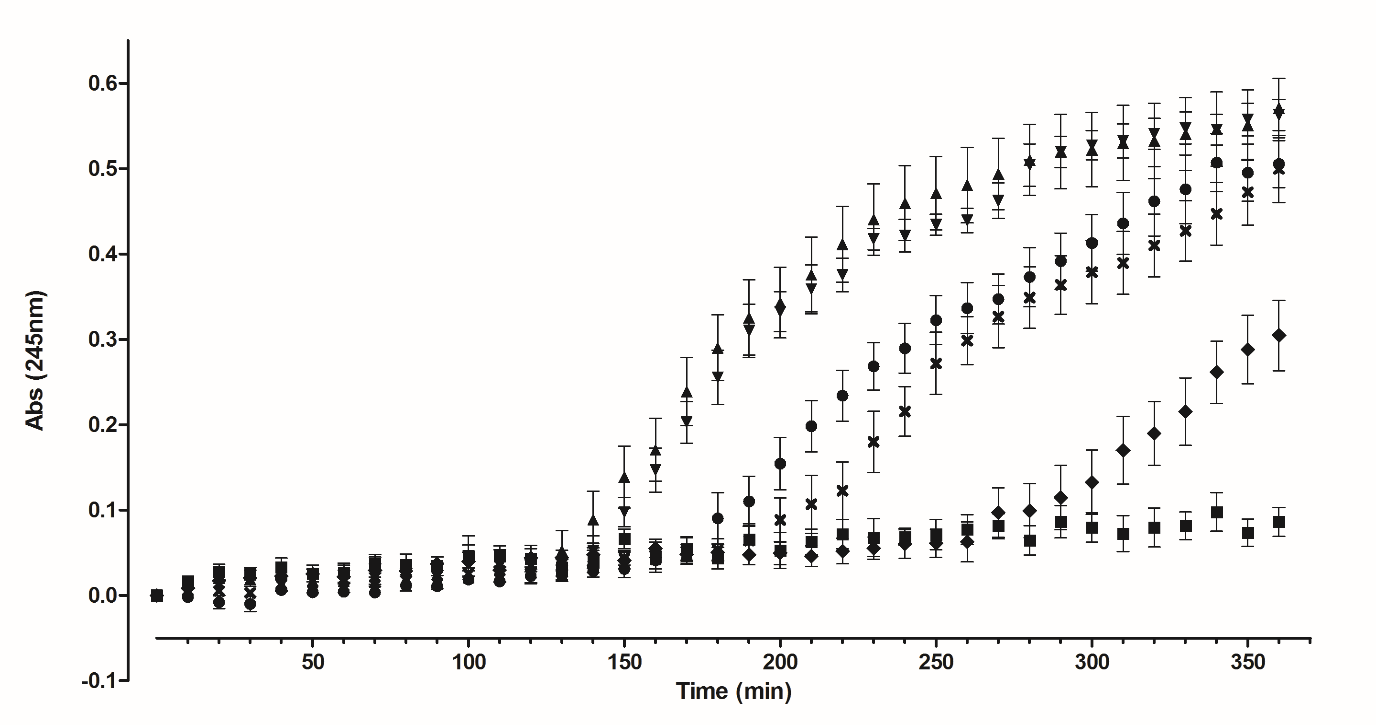

Supplement: Additional file 1: Figure S1. — Copper-induced oxidation curves of human serum samples with increasing concentrations of BME w/v (▲; Control, ▼; 0.0625%; ●; 0.125%; x; 0.25%; ♦; 0.5% and ■; 0.75%). Data are shown as mean ± SEM of n = 6. (TIF 135 kb) [file 12906_2016_1522_MOESM1_ESM.tif]
