# Supplementary material for: Bitter melon protects against ER stress in LS174T colonic epithelial cells
Source: BMC Complement Altern Med. 2017 Jan 3;17:2. doi: 10.1186/s12906-016-1522-1 (PMC5210302; doi:10.1186/s12906-016-1522-1)
Supplement: Additional file 2: Table S1. — Effect of increasing concentrations of BME on copper-induced oxidation of human serum. (DOCX 12 kb) [file 12906_2016_1522_MOESM2_ESM.docx]

**Supplementary Table 1:** Effect of increasing concentrations of BME on copper-induced oxidation of human serum.

| Concentrations | Lag time (min) | Rate of Oxidation (Δ Abs_245_/Δ min) | Maximum Δ in Abs_245_ |
| --- | --- | --- | --- |
| Vehicle | 144 ± 15 | 0.0059 ± 0.0003 | 0.570 ± 0.035 |
| BME 0.0625% | 162 ± 18 | 0.0051 ± 0.0003 | 0.564 ± 0.018 |
| BME 0.125% | 212 ± 16* | 0.0059 ± 0.0002 | 0.506 ± 0.028 |
| BME 0.25% | 254 ± 14* | 0.0049 ± 0.0004 | 0.490 ± 0.038 |
| BME 0.50% | 283 ± 30* | 0.0054 ± 0.0001 | 0.295 ± 0.045* |
| BME 0.75% | >360 | nc | 0.079 ± 0.009* |

Values are presented as mean ± SD of *n* = 6. *p < 0.05 compared to control (no BME) after adjusting for multiple comparisons. nc = not calculated
